# Patients’ perception of privacy and confidentiality in the emergency department of a busy obstetric unit

**DOI:** 10.1186/s12913-018-3782-6

**Published:** 2018-12-18

**Authors:** Lucia Hartigan, Leanne Cussen, Sarah Meaney, Keelin O’Donoghue

**Affiliations:** 1 Pregnancy Loss Research Group, Department of Obstetrics and Gynaecology, University College Cork, Cork, Ireland; 2 Cork University Maternity Hospital, Wilton, Cork, Ireland; 3 National Perinatal Epidemiology Centre, University College Cork, Cork University Maternity Hospital, Wilton, Cork, Ireland; 4 The Irish Centre for Fetal and Neonatal Translational Research (INFANT), University College Cork, Cork, Ireland

**Keywords:** Emergency department, Privacy, Confidentiality, Maternity unit, Pregnancy loss

## Abstract

**Background:**

Privacy and confidentiality are central components of patient care and are of particular importance in obstetrics and gynaecology, where clinical situations of a sensitive nature regularly occur. The layout of the emergency department (ED) in maternity units is often not conducive to maintaining privacy.

**Method:**

Our study aimed to discover if changing the environment could improve patients’ experiences in the ED. We surveyed patients and asked specific questions about their perception of privacy in the ED. We then repeated the survey following renovations to the ED which involved replacing curtained patient areas with walled cubicles.

**Results:**

There were 75 pre-renovation surveys and 82 post-renovation surveys completed. Before the renovations took place, only 21% (*n* = 16) found their privacy to be adequate during their visit to the ED. However this rose to 89% (*n* = 73) post-renovation.

**Conclusion:**

Our study showed that patients’ perception of privacy and confidentiality significantly improved following refurbishment of the ED.

**Electronic supplementary material:**

The online version of this article (10.1186/s12913-018-3782-6) contains supplementary material, which is available to authorized users.

## Background

Privacy and confidentiality are critically important components of patient care [1, 2]. Healthcare professionals are ethically obliged to protect patient confidentiality and ensure discretion [3, 4]. The patient’s rights to privacy and confidentiality of their health information should be respected in all settings [1]. This should include informal situations, for example corridor conversations and social settings [1]. Overheard disclosures adversely affect patients’ trust and can lead to a breakdown in the relationship between them and their healthcare team [5].

The Emergency Department (ED), by its nature, can have a chaotic atmosphere making it difficult for staff to create a suitable environment in which to provide care. Privacy and confidentiality breaches in emergency departments are commonplace [6]. A number of studies have examined privacy and confidentiality in the ED. One US study observed that all members of the healthcare team violated patient confidentiality during the normal process of patient care [6]. The same study found that the physical layout of the ED affected confidentiality and privacy. The authors reported that “curtained walls” led to frequent breaches of privacy and confidentiality.

Further, another US study reported that compared with rooms with walls, patients who were treated behind curtains more often believed that they could overhear others and that others could hear them, view them, hear personal information, and view personal parts of their bodies [7]. More recently Australian authors reported that 41% of surveyed patients overheard others’ conversations with staff. As was the case in other studies, those in walled cubicles experienced fewer privacy breaches than those in curtained cubicles [8].

Maternity unit EDs are busy locations, where it can prove difficult to provide the level of sensitivity that the common clinical presentations in Obstetrics and Gynaecology require. Many maternity units, for practical reasons, use one hospital location to assess all types of emergencies. These include early pregnancy pain or bleeding, women presenting in labour or with complications in pregnancy, as well as non-pregnant women with gynaecological complaints. Therefore, a woman who has just suffered a miscarriage, for example, might be cared for in the cubicle next to a woman who is in the early stages of labour. Joy and sadness can be commonly juxtaposed in the ED of a maternity unit, making it a unique setting where privacy is of particular importance.

The Irish Standards for Bereavement Care following Pregnancy Loss and Perinatal Death, published in 2016 advocate patient-centred care and recommend that hospitals facilitate access to spaces where delivering bad news and bereavement care can take place in a quiet, comfortable environment, where privacy is ensured [9]. Intuitively, it is imperative to maintain a woman’s dignity during her attendance at a maternity unit ED, yet there is a paucity of literature dealing specifically with matters of privacy and confidentiality in these settings.

In our hospital, it was recognised that ED presentations were a significant source of patient complaints and that a significant portion of these complaints were related to lack of privacy and confidentiality. A previous study from our hospital, which focused on patient experiences of miscarriage, highlighted how negative experiences were often related to the physical design of the hospital [10]. These participants felt that the physical space where they were cared for in the ED heightened their distress [10].

We aimed to examine if changing the physical layout of the maternity ED, with some simple refurbishments, would improve the patient experience of privacy and confidentiality. This study aimed to assess patients’ perception of privacy and confidentiality during their visit to the ED and whether or not patients’ experiences altered after the physical layout of the ED changed.

## Methods

Cork University Maternity Hospital (CUMH) is a university teaching tertiary referral maternity hospital in the south of Ireland where over 8000 babies are delivered annually. Maternity care in this hospital is consultant-led but involves a multi-disciplinary team including non-consultant hospital doctors, midwives, physiotherapists, chaplains and care assistants. The ED at CUMH is a 24-h service, and is the first point of access for all women utilising the services of the hospital. There are approximately 17,000 attendances in the ED annually and this comprises early pregnancy, antenatal, postnatal and gynaecology patients. Originally, the physical layout of the CUMH ED afforded no partitioning for women attending, with only a curtain separating each of the five trolley bays in the unit.

**Fig. 1:**
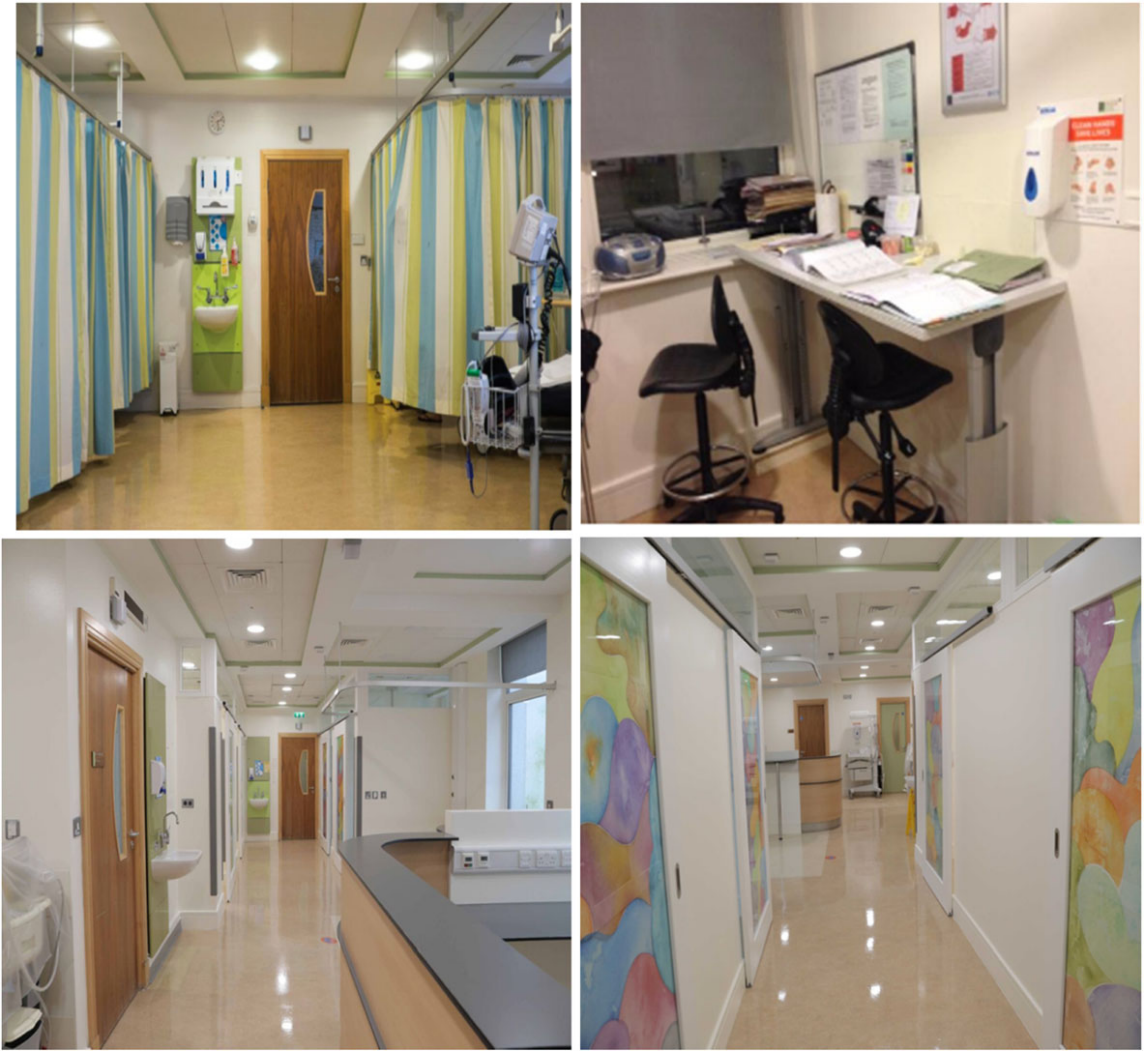
Photographs of the ER at Cork University Maternity hospital before and after a refurbishment project which was undertaken to achieve a more private and suitable space in which to care for patients. Top right and left: pre- renovations. Bottom left and right: post-renovations

In 2015 a decision was made to improve the CUMH ED layout, as it was realised that privacy and confidentiality was compromised by its physical design. The objective of the refurbishment project was to create an improved environment with individual walled cubicles within the ED, to improve the experience of patients being assessed, examined, and/or admitted. The nurses’ station was repositioned so that it would be further away from the patient care areas. Figure 1 contains photographs from before and after the refurbishment project.

Our study was questionnaire-based and asked women specific questions about their perception of privacy and confidentiality during their assessment in the ED. The survey, which is displayed in Additional file 1, was created by the authors, specifically developed for this study and based on previously published literature for the general ED setting. Olsen et. Al used a questionnaire which asked patients if they had “overheard doctors/nurses conversations about myself or other patients” and if they had “overheard inappropriate or unprofessional comments by staff” [11]. We elaborated on these questions in our questionnaire and created questions appropriate for a maternity setting and for our specific hospital environment. Women were asked:

- if they had overheard a conversation about themselves or another patient,
- where the conversation had taken place: the cubicle beside them, at the midwives’ station, or if someone was talking about them on the phone,
- what type of information women had overheard, such as personal information, about their symptoms, their medical history or their test results,
- or had they overheard staff speaking about non-medical matters such as their own personal lives.

Finally, women were asked to comment on whether or not they deemed their privacy to be adequate and given the opportunity to comment on anything in particular that they saw or heard which upset them during the time that they were in the ED. Tables within the results section contain a sample of these comments which were chosen by the authors as they best illustrate the themes being presented.

Informed verbal consent was obtained from participants following approval from the local ethics committee in CUMH. Verbal consent was deemed to be appropriate as the surveys were completely anonymous with no patient identifiable details. Patients were under no obligation to complete the surveys if they did not wish to partake in the study.

Women were asked to complete an anonymous survey during a visit to the ED over a 4 week period before the refurbishment project took place. Then, some weeks after the refurbished unit opened, a separate group of women were surveyed using the same questionnaire. The latter took place over another 4 week period.

GPower 11 was used to determine the sample size for the Chi-Square tests, which determined that it was necessary to recruit approximately 71 women to achieve statistical power > 90% using the standard 5% level of statistical significance. All healthcare staff who work in the ED were educated about the study, were familiar with the survey and agreed to distribute it to patients during their attendance or immediately prior to discharge from the ED. Staff were asked to randomly select patients to participate both during the day and night. The surveys were in paper format and were handed directly to patients by midwives and doctors working in the ED. Participants were asked to place their completed surveys in a sealed letter box when they were leaving the ED having either been admitted to the ward or discharged home. A number of patients would have been deemed ineligible to participate, for example, a patient who was very upset following the diagnosis of a stillbirth or in the setting of a collapse. Staff used their own discretion as to who it was inappropriate to approach.

Following collection of the surveys, data were inputted and analysed using SPSS version 20.0. The pre- and post-intervention surveys were analysed separately using descriptive statistics. The differences between pre- and post-intervention surveys were then compared using Chi-square tests. Qualitative data were collected from the open-ended questions to identify themes and sub-themes. For the purposes of this study, we took an idiographic approach which meant that themes emerged from the data as it was analysed. Analysis took place in phases- we firstly familiarised ourselves with the data, then we searched for initial sub-themes and finally defined and named our themes. Given the number of transcripts that needed to be managed for this analysis it was agreed that software for analysis was not necessary. Therefore, the analysis was undertaken using Microsoft word.

## Results

In total, 157 surveys were completed and included in data analysis. These comprised 75 pre-renovation and 82 post-renovation surveys. The majority, 86% (*n* = 135) of those surveyed were pregnant, with the remaining 14% (*n* = 22) comprising post-natal or gynaecology patients. Before the renovations took place, only 21% (*n* = 16) women found their privacy to be adequate during their visit to the ED, however this rose to 89% (*n* = 73) in the post renovation survey which is a highly significant finding (*p* < 0.001).Women were given the opportunity to comment on whether or not they saw or heard anything that upset or disturbed them during their visit to the ED. Table 1 contains comments from those surveyed before refurbishment took place and displays the theme of an unsatisfactory patient experience with sub-themes of disappointment, disgust, invasion of privacy, loss of dignity and dissatisfaction.

**Table 1:** As part of the questionnaire used in this study, women were given the opportunity to comment on whether or not they saw or heard anything that upset or disturbed them during their visit to the emergency room (ER) of Cork University Maternity Hospital (CUMH). This table outlines some of the comments from those surveyed before refurbishment took place

Theme	Subcategories	Illustrative Quotes
Unsatisfactory patient experience	Anxiety Disappointment Dissatisfaction Disgust Invasion of privacy Loss of dignity	“Very busy; Not very private”.
		“I was in a cubicle by the main door and the curtain wasn’t fully closed so that people passing by could see in”.
		“I was examined behind a curtain and at one point, men and other patients could see me and it made me very uncomfortable”.
		“I could see blood on the ground in the cubicle beside me”.
		“I could overhear absolutely everything while I was in the Emergency Room”.
		“I overheard nurses answering queries over the phone”.
		“I was upset by seeing other people in severe pain near me”.
		“Through a gap in the curtains I saw another patient’s heavily soiled/bloody sanitary towel being carried to the bin by a member of staff”.

Almost half, 49% (*n* = 37) of the women surveyed pre-renovation admitted to overhearing a conversation about themselves during their visit to the ER whereas post-renovation only 11% (*n* = 9) reported this experience (*p* < 0.001). Similarly, 49% (*n* = 37) of the patients who were surveyed pre-renovation also had overheard a conversation about another patient while in the ED and this fell to 9.8% (*n* = 8) (*p* < 0.001) following refurbishments.

Table 2 outlines comments from those surveyed after the renovation project. Sub-themes of appreciation, patient satisfaction, privacy and dignity are presented here. These comments were considerably more positive, suggesting an overall improved and more satisfactory patient experience. The contrast between women’s comments pre and post renovation, clearly indicate that privacy is greatly improved when doors replace curtains in the ED setting. It is important to highlight, however, that doors do not eliminate privacy breaches entirely. Table 3 contains comments made by women post-refurbishment and emphasise the importance of discretion at all times when caring for patients.

**Table 2:** As part of the questionnaire used in this study, women were given the opportunity to comment on whether or not they saw or heard anything that upset or disturbed them during their visit to the emergency room (ER) of Cork University Maternity Hospital (CUMH). This table outlines some of the comments from those surveyed after refurbishment took place

Theme	Subcategories	Illustrative Quotes
Satisfactory patient experience	Patient satisfaction Appreciation Positive patient experience	• “Nothing I heard troubled or disturbed me”
		• “I was in ER twice in the last 10 days and both times were grand and private”.
		• “Everyone was so helpful and well-mannered although they were all so busy”.
		• “Doctors in ER were very responsive and efficient”.
		• “Highly professional attitude; extremely happy with my experience”.
		• “The nurses and doctors couldn’t do enough for me”.

**Table 3:** This table outlines some of the negative comments after refurbishment took place highlighting that walled cubicles do not completely eliminate the risk of privacy breaches and the importance of upholding discretion and professionalism at all times when caring for patients

Illustrative Quotes	
“When I was being examined, the nurse did not fully close the door and I felt exposed”.	
“The doctor that was called to review me opened the door without knocking while I was having an internal exam. The other doctor who was examining me quickly tried to cover me up but I was on view to the outside”.	
“I could hear the staff discussing my scan results at the nurses’ station”.	

## Discussion

We asked women about their perception of privacy and confidentiality as they attended our ED when there were curtained cubicles separating the trolleys. After renovations, which made simple but strategic changes to the layout of the ED, we repeated the survey.

Following refurbishment of the ED, we found that there was significant improvement in women’s perception of their overall privacy and confidentiality when they were assessed and treated in individual walled cubicles compared with curtained cubicles. (89% vs. 21%) Half of those surveyed pre-renovation admitted to overhearing a conversation about themselves or another patient. This fell to 11 and 9.8% respectively post-renovation. Our study shows that simply changing the layout or design of the ED in a maternity unit can impact favourably on the patient experience**.**

Our findings echo the results of studies from general EDs which conclude that privacy and confidentiality breaches are routine occurrences in emergency rooms [3]. As with other studies, our findings suggest that privacy is improved with walled cubicles compared with curtains [3, 5].

Our study is unique because, to the authors’ best knowledge, it is the first to look specifically at privacy and confidentiality breaches in a maternity ED. Studying this in maternity settings is important because clinical situations of a particularly sensitive nature occur on a regular basis. In the ED of a maternity unit, a woman could be experiencing a traumatic pregnancy loss or receiving a serious diagnosis while listening to a fetal heartbeat recording or conversations nearby.

The publication of The Irish Standards for Bereavement Care following Pregnancy Loss and Perinatal Death in 2016 recommend that breaking bad news should be done in a comfortable, quiet environment where privacy is ensured [11]. This guidance, published subsequent to our study, further emphasises the relevance and importance of our project. We also know of at least one other maternity unit in Ireland who, prompted by the findings of our project, have undertaken renovation of their ED.

Previously, most of the available literature has compared patients cared for in different environments (i.e. walls or curtains) within the same setting or department whereas, this study looked specifically at perceptions of privacy and confidentiality before and after a refurbishment project. Moreover, most other investigators did not ask patients to comment on whether or not they were upset by something they had seen or overheard. The contrast between the pre- and post-renovation comments outlined in Tables 1 and 2 corroborate the significant results and indicate that simple renovations can improve patients’ perception of privacy and confidentiality.

Our questionnaires were anonymous which is likely to have promoted honest responses from women. Patients completed the questionnaires during or shortly after their attendance in the ED, which removes the limitation of recall bias. We acknowledge that as the questionnaires were only produced in the English language, this might have deterred women who do not speak English as their first language from completing the surveys. However, among the responders, there were nine distinct nationalities represented, reflecting the typical ethnic demographics of the patient population attending our unit.

As with any survey, responses may not really reveal true views. Although measures were used to minimise bias, patients may have declined to give unfavourable responses to survey questions. There were some privacy issues that were not addressed, such as management of personal data and medical notes. Further limitations of our study include relatively small numbers, participants were randomly selected by staff working in the ED and that, due to the nature of pregnancy, it was not possible to survey the same group pre and post refurbishment.

## Conclusions

In conclusion, our study indicates that the refurbishment of the ED in our maternity unit has improved privacy and confidentiality for the women attending. We recommend that obstetric units consider walled rooms instead of curtained cubicles when renovating or designing new ED departments in order to protect patient privacy and confidentiality and improve the quality of the patient experience.

## Additional file

Additional file 1: This is the questionnaire that was used in the study. (DOCX 43 kb)

## Abbreviations

CUMH: Cork University Maternity Hospital
ED: Emergency Department
