# Supplementary material for: Patients’ perception of privacy and confidentiality in the emergency department of a busy obstetric unit
Source: BMC Health Serv Res. 2018 Dec 18;18:978. doi: 10.1186/s12913-018-3782-6 (PMC6299575; doi:10.1186/s12913-018-3782-6)
Supplement: Supplementary file 1 — This is the questionnaire that was used in the study. (DOCX 43 kb) [file 12913_2018_3782_MOESM1_ESM.docx]

**Privacy, Dignity and Confidentiality in the Emergency Room (ER) at CUMH**

***We are asking those who attend the emergency department about issues of privacy and confidentiality.***

**SECTION A. THIS IS ABOUT YOU.**

**How old are you?** **What is your nationality?**

I am _______ years old ___________________________

**Please indicate whether you are:**

Pregnant  Not Pregnant

**Are you a private patient of a particular consultant?** (Please tick *one* box)

Yes  No

**Were you referred to the Emergency Room (ER) by your GP?** (Please tick *one* box)

Yes  No

**SECTION B. THIS IS ABOUT YOUR VISIT TO THE ER TODAY.**

**1. During my current visit to the ER I overheard a conversation about myself.**

Yes  ^Go to Q1a^ No  ^Go to Q2^

Q1a. If you answered yes, please indicate where the conversation took place? (Please tick all that apply)

- Cubicle beside me
- At the Nurses’ Station
- Someone was talking about me on the phone
- Unsure exactly where

Q1b. What type of information did you overhear?

(Please tick all that apply)

- My personal details
- My symptoms/reasons for coming to the ER today
- My medical history
- My test result (for example, blood test results or scan findings)

**2. During my current visit to the ER, I overheard a conversation about another patient.**

Yes  ^Go to Q2a^ No  ^Go to Q3^

Q2a. If you answered yes, please indicate where the conversation took place? (Please tick all that apply)

- Cubicle beside me
- At the Nurses’ Station
- Someone was talking about them on the phone
- Unsure exactly where

Q2b. What type of information did you overhear? (Please tick all that apply)

- Another patient’s personal details
- Another patients’s symptoms/reason for coming to the ER today
- Another patient’s medical history
- Another patient’s test result (for example, blood test results or scan findings)

**3. During my current visit to the ER, I overheard medical or midwifery/nursing staff chatting about non-medical matters (for example, their personal or private lives).**

Yes  No

**4. The privacy of my room or cubicle in the ER was adequate**

Yes  No

**5.** **Did you hear or see anything else that troubled or disturbed you on this visit to the Emergency Room in CUMH? Please tell us.**

**_________________________________________________________________________________________________________________________________________________________________________________________________________________________________**

***All information will remain anonymous.  Thank you for participating in this survey.***
